# Exposure of cells to near-infrared irradiation relaxes chromatin compaction and facilitates recognition of cyclo-butane pyrimidine dimers

**DOI:** 10.1038/s41598-025-08763-z

**Published:** 2025-07-01

**Authors:** Beata Plitta-Michalak, Ksenia G Kolobynina, Qinghua Qin, Ishita Jain, I-Peng Chen, Stefan Henning, Beate Volkmer, Rüdiger Greinert, Anja Tham, Petra Boukamp, Alexander Rapp

**Affiliations:** 1 Cell Biology and Epigenetics, Department of Biology, Technical University of Darmstadt, Schnittspahnstr. 10, Darmstadt, 64287 Germany; 2 Centre of Dermatology, Elbe Clinics, Am Krankenhaus 1, Buxtehude, 21614 Germany; 3 German Cancer Research Center (DKFZ), Im Neuenheimer Feld 280, Heidelberg, 69120 Germany; 4 Present Address: Department of Chemistry, Faculty of Agriculture and Forestry, University of Warmia and Mazury, Plac Łódzki 4, Olsztyn, 10-957 Poland

**Keywords:** Chromatin relaxation, Ultraviolet radiation, Cyclo-butane pyrimidine dimer, Near infrared radiation, DNA repair, Nuclear organization, Natural hazards, Risk factors

## Abstract

Ultraviolet A and B (UVA 320–400 nm and UVB 280–320 nm) induced cyclobutane-pyrimidine dimers (CPDs) are the most critical lesions caused by environmental sun exposure. Here we show that CPD removal is accelerated when, in addition to UV, cells are simultaneously exposed to water-filtered near-infrared (nIR, 750–1600 nm). The described effect is dose-dependent on the nIR-dose and is found in skin keratinocytes and fibroblasts. Accelerated removal of CPDs, which coincides with chromatin relaxation and faster CPD recognition, occurs after nIR exposure. While nIR alone does not affect cellular survival, co-exposure to UVB leads to reduced cellular survival and an increased number of mutations. Increasing single strand break levels (SSB) occur transiently after nIR exposure and independent of reactive oxygen species (ROS) formation. These data suggest that the rate-limiting step in the NER repair process – damage recognition – is facilitated by nIR-induced chromatin relaxation, causing the accumulation of unnatural high levels of SSBs and single stranded DNA, unfavourable for the cell fate resulting in reduced survival and increased mutation rates. Since nIR modulates the UV-dependent damage response, risk estimation of solar radiation-induced DNA damage should not only consider the UV components but also include the nIR fraction of the solar spectrum.

## Introduction

While UVC radiation (< 280 nm) is absorbed by the atmosphere, UVB- (280–320 nm) and UVA- (320–400 nm) radiation, in addition to visible and infrared light, are present in natural sun light reaching earth`s surface (with infrared light representing the largest fraction)^1^. Importantly, skin cancer induction is causally linked to UV-exposure however, the exposure modus varies for the different skin cancer types. Accordingly, malignant melanoma (MM) develops after *intermittent* exposure (especially in early adulthood), squamous cell carcinoma (SCC) after *cumulative (lifelong)* exposure, and basal cell carcinoma in dependence of *cumulative and intermittent* exposure^2–4^.

Among the various effects of UV exposure, the induction of CPDs in cellular DNA has the strongest impact, since CPDs are both mutagenic and cytotoxic^5^. CPD induction occurs predominantly by UVC (naturally not relevant on Earth’s surface) or UVB exposure since the direct absorption of these photons by DNA is most efficient for the formation of CPDs. Additionally, UVA was reported to induce CPDs, although at a significantly reduced rate^6,7^. UV-induced DNA lesions do not occur evenly or randomly generated throughout the nuclear DNA. Both the position of the DNA within the nucleus^8^as well as it’s position relative to the nucleosome^9^ are important factors that affect DNA damage induction by UV as well as cellular repair of UV-induced DNA lesions. The DDB1-DDB2 complex performs an essential function in the initial detection of photo-lesions both in DNA bound to the nucleosome and the linker DNA^10^.

CPDs, as other bulky adducts, are repaired in the cell by the nucleotide excision repair (NER) pathway^11,12^. This pathway allows for two different options: the transcription-coupled NER (tc-NER) and the global genomic NER (gg-NER). Both repair-pathways include the same enzymatically supported steps: damage recognition, damage verification, double strand opening, excision of damaged strand and re-synthesis. Damage recognition is a rate limiting step^13,14^. In tc-NER the damage is recognized when RNA polymerase encounters a damaged base and is therefore blocked from ongoing transcription. This initiates the recruitment of CSB and subsequently initiates the tc-NER. In non-transcribed regions the damage is sensed by the UV-DDB complex, consisting of DDB1 and DDB2. Binding of UV-DDB was reported to precede XPC binding^15^ and that photo-lesions cause activation of the associated ubiquitin ligase CUL4A and the subsequent ubiquitination of proteins around the damage site^10^. Local chromatin relaxation is an effect of chromatin ubiquitination around the damage site^16^. XPC was shown to scan the DNA^17,18^ and when a damage is encountered the gg-NER is initiated^12,19^. Chromatin compaction affects the scanning process since efficient damage recognition by XPC relies on the accessibility of the damage^19,20^. Therefore, more bulky NER substrates, such as benz-a-pyrene or 6-4-photoproducts (another, less frequent, UV DNA-lesion) are more efficiently detected (and repaired) compared to CPDs^21^ which are less bulky and more hidden in the DNA helix structure and are only inducing minor helix distortions^22^. Recognition of UV-photo-products through/by XPC is supported by the UV damage binding complex (UV-DDB complex)^23^an ubiquitin ligase associated complex, supposed to locally de-compact chromatin^24^. Emerging knowledge has been gained on the response of chromatin upon induction and subsequent repair of CPDs. Especially the steps of de-compaction, damage processing, and re-compaction are essential for effective DNA repair^9,19,25^. Similar to X-ray induced damage, chromatin de-compaction was reported for the repair of CPDs, where chromatin compaction is transiently uncoupled from its epigenetic control mechanisms (e.g. histone modifications), allowing access to the damage sites and processing of DNA repair, before restoring the (“pre-damage”) epigenetic marks of the chromatin^26–30^.

Currently, most studies on the phototoxicity of CPDs are conducted using specific light sources, primarily in the UVB or even UVC spectrum. Since the natural exposure is different with the sun spectrum containing both UVA and UVB in addition to visible and near infrared light, a more realistic scenario would require studying the complete optical solar spectrum (UVA + UVB + visible + infrared light). These investigations must include the simultaneous irradiation with defined spectral components to allow the study of possible interactions.

Until today only a limited number of studies are available, which use defined spectral bands (e.g. UVB, UVA, nIR and/or combinations of these bands) to assess their impact on photo-damage induction and repair^31–33^. Especially the near infra-red (nIR) spectrum was reported to have opposing effects in combination with other spectral ranges on the induction and repair as well as on the cellular response^34–36^. When nIR effects are studied, it is important to separate thermal from non-thermal effects. Even in combination with X-ray exposure an effect of pre-irradiation with nIR was reported to affect the cellular repair^37^. Similarly, pre-exposure of cells to nIR was reported to reduce UV-induced toxicity^38^. Among possible non-thermal effects of nIR, the generation of reactive oxygen species (ROS) and “DNA vibration” are discussed^39,40^. This is reported together with further effects of nIR on skin such as skin tightening, due to nIR interaction with elastin and collagen and beneficial effects on wound healing^39,41^. On the other hand enhanced tumorigenicity was reported, but this is described to be an effect of excessive heat production^42^.

In order to get a better understanding of individual spectral bands of solar radiation in comparison to the entire solar spectrum, we designed an irradiation device that is able to independently deliver solar spectral components (UVB, UVA, visible light and water-filtered nIR) independently or as any mixture of the four. Thereby, it is generating an exposure situation closer to the environmental solar exposition scenario^43^. In previous studies we showed that the effect of combined spectral exposure on cell survival, cell cycle alterations and metabolic activity is not reflected by a simple additive effect of the individual spectra, but that combined spectra have different effects^43^. We therefore aimed to study the impact of the full solar spectrum on the induction and repair of CPDs as well as the combinations of the individual spectral components in detail, with the crucial point being to evaluate the role of nIR in DNA damage induction and repair.

## Results

### UVA and UVB induce CPDs

**Fig. 1:**
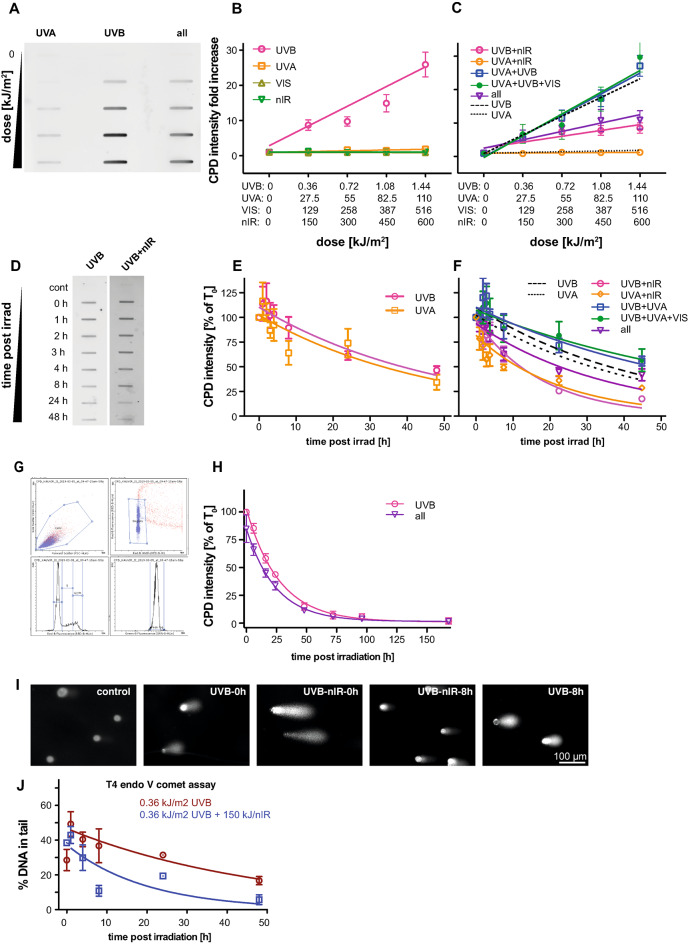
CPD induction and repair after exposure to different spectra. **(A)** Sample immune-slot blot to quantify CPD induction following increasing doses of UVA and UVB as well as a combination of all spectra (UVB, UVA, VIS and nIR). (**B)** Quantification of induced CPDs after exposure towards individual spectral bands. A linear dose-dependency between UVB dose and CPD induction was found. A very weak induction was found after UVA exposure also (see Supplementary Fig. 1). (**C)** As in (**B)** but for all possible combination of the four spectral bands. The dashed line represents the induction by UVB only, while the dotted line represents the induction by UVA only, as shown in Fig. 1**B**. (**D)** Removal of CPDs from genomic DNA was measured by immuno-slot blot. Example showing two typical slot blots. (**E)** Measurements of CPD removal induced by either UVB (pink) or UVA (orange) exposure. The resulting intensities were normalized to the intensities directly after exposure (time 0) and fitted with a single exponential decay function. (**F)** Analogue to **(C)** the CPD removal after the exposure to combined spectra is shown. The dashed black line again represents the exponential decay fit function from (**E**) for UVB and the dotted for UVA respectively. The plotted values represent the mean of at least 3 independent measurements; the error bars represent the standard error of the mean. (**G)** Measuring CPD removal by flow cytometry. The gating schema is based on forward (FSC) scatter and side scatter (SSC), followed by gating for single cells based on the DNA quantification. From the resulting DNA content histogram only the G1 cell equivalent was used for quantification. (**H)** Quantification of CPD levels for either UVB alone or the complete spectrum (UVB, UVA, VIS, nIR). Datapoints represent mean values of at least three replicates. Error bars represent the standard error of the mean. Decay curves were fitted with a single exponential function. (**I)** Sample images of the comet assay. Shown are comets from untreated controls, UVB irradiated cells directly and 8 h after irradiation, and cells irradiated with the combination of UVB and nIR directly and 8 h post irradiation. The lysed cells were incubated with T4 endo VII endonuclease to convert CPDs to strand breaks. (**J)** Quantification of the comet assay. The comets were analysed in terms of DNA in tail and the residual CPDs were quantified as migrated DNA. Each datapoint represents the mean of means from three biological replicates with at least 50 scored comets per slide. Error bars are the standard error of the mean.

First, we established the dose- and radiation quality-dependent induction of CPDs in genomic DNA in exponentially growing HaCaT cells by isolating total genomic DNA and quantifying the CPD amount by genomic immuno-slot-blots. Figure 1 A shows an example slot blot (all full blots together with the quantification are given in Supplementary Information S1). Quantification of the band intensities revealed a dose-dependent induction of CPDs by UVB (Fig. 1B). Also, for UVA a dose-dependent, although much smaller, induction of CPDs could be observed (Figure S1A and B). For the isolated visual (VIS) spectrum, as well as for the nIR spectrum (both measured individually), we did not find a significant induction of CPDs (Fig. 1B).

### CPD induction by UVA and UVB exposure is modulated by nIR co-exposure

Next, we analyzed the induction of CPDs when combined spectra were applied (Fig. 1C). UVB in combination with UVA (blue squares) or in combination with UVA and VIS (green dots) all showed a similar or even slightly elevated, although not significant, induction as UVB alone (black dashed line), the combination of nIR and UVB (pink dots) as well as the combined full spectrum (purple triangles), however, showed significantly reduced levels of CPD induction. The combination of UVA with nIR (yellow circles) also exhibited reduced amounts of CPDs, although at a very low, non-significant level, compared to UVA alone (dotted black line). To ensure that the observed effect is not unique to HaCaT cells, we conducted similar experiments using primary human dermal fibroblasts. Also in these cells, we found the reduction of CPDs when UVB irradiation was applied together with nIR (Figure S1C and S1D, green triangles), as compared to cells exposed to UVB only (purple dots). The slope for CPD induction was reduced by approximately 2.5 fold when UVB was combined with nIR co-exposure.

### CPD removal is accelerated when nIR is co-exposed

Since, under our experimental conditions, irradiation of cells requires exposure times of 10 to 40 min, we speculated that the reduced CPD levels after UVB + nIR, UVA + nIR and exposure to all radiation spectral components (see Fig. 1C) might be due to CPD repair already during the exposure time. Therefore, we performed measurements of CPD repair kinetics with the lowest doses for the initial CPD induction (360 J/m^2^ UVB equivalent as used in Fig. 1B and C) and quantification was normalized to time point 0 (directly after the end of the exposure = 100%). Repair kinetics were fitted using single exponential decay functions and half-life time was determined (ln(2)/K). Repair kinetics for UVB and UVA spectra (applied individually) are shown in Fig. 1E. CPD induction by both spectra showed comparable repair kinetics with half-life times Ƭ=33.9 h for UVB and Ƭ=32.4 h for UVA, respectively. Since we did not find induction of CPDs for VIS and nIR we did not perform the repair kinetics for these exposures.

Then, we again combined different spectra and measured the CPD removal accordingly. When UVB was combined with UVA or with UVA and VIS simultaneously, we found a delay in CPD removal compared to UVB exposure alone (Fig. 1F, blue squares and green dots compared to the dashed black line). The half-life time of CPD persistence increased from Ƭ= 33.9 h to Ƭ = 53.0 and Ƭ = 44.8 h, respectively. In contrast, when UVB exposure was either combined with nIR (pink circles) or the full spectrum (purple triangles) half-life time was reduced from Ƭ = 33.9 to Ƭ = 13.5 and Ƭ = 26.8 h, respectively. A similar effect could be observed when the repair of UVA induced CPDs was analyzed. Here, the persistence of CPDs was reduced from Ƭ = 32.4 h (dotted black line) for isolated UVA exposure to Ƭ = 17.2 h for UVA combined with nIR (yellow diamonds).

To verify these findings, we used flow cytometry to quantify the CPD levels. In these experimental series, the cells were exposed as described above and fixed at the indicated time points. The CPD amounts were quantified after immuno-fluorescence staining for CPDs by flow cytometry in G1 cells for up to 7 days. Figure 1G and H show the repair kinetics of UVB, and the complete spectrum (UVB, UVA, VIS and nIR). While isolated UVB exposure resulted in a repair half-life time for CPDs of Ƭ = 19.4 h the co-exposed cells (complete spectrum) showed a reduced CPD half-life time of Ƭ = 17.7 h.

To confirm the nIR effect independently of antibody staining, we used the modified alkaline comet assay^44^. In this assay, the cells were embedded and lysed. However, before electrophoresis the gels/cells were incubated with T4 endonuclease V, which specifically recognizes CPDs and incises the DNA, thus converting CPDs to strand breaks^45^. With this method, which allows to measure CPD quantities on a single cell level, we also detected an enhanced removal of CPDs from genomic DNA when cells were irradiated with a combination of UVB and nIR (Fig. 1I-J). These measurements demonstrated a reduction of the CPD half-life time from Ƭ = 34.1 h for UVB to Ƭ = 13.8 h for cells that were irradiated with the combination of UVB and nIR. Taken together, we showed with three independent methods (all summarized in Table 1) that co-exposure of nIR with UVB leads to an accelerated removal of CPDs from genomic DNA.

**Table 1:** CPD repair kinetics depending on the method and irradiation regime.

Method/Irradiation	UVB	UVB nIR	UVA	UVA nIR	UVB UVA	UVB UVA VIS	UVB UVA VIS nIR
Slot blot	33.9 h	13.5 h	32.4 h	17.2 h	53.0 h	44.8 h	26.8 h
Flow cytometry	19.4 h						17.7 h
Comet-assay	34.1 h	13 0.8 h					

### Acceleration of CPD removal is dependent on nIR doses

**Fig. 2:**
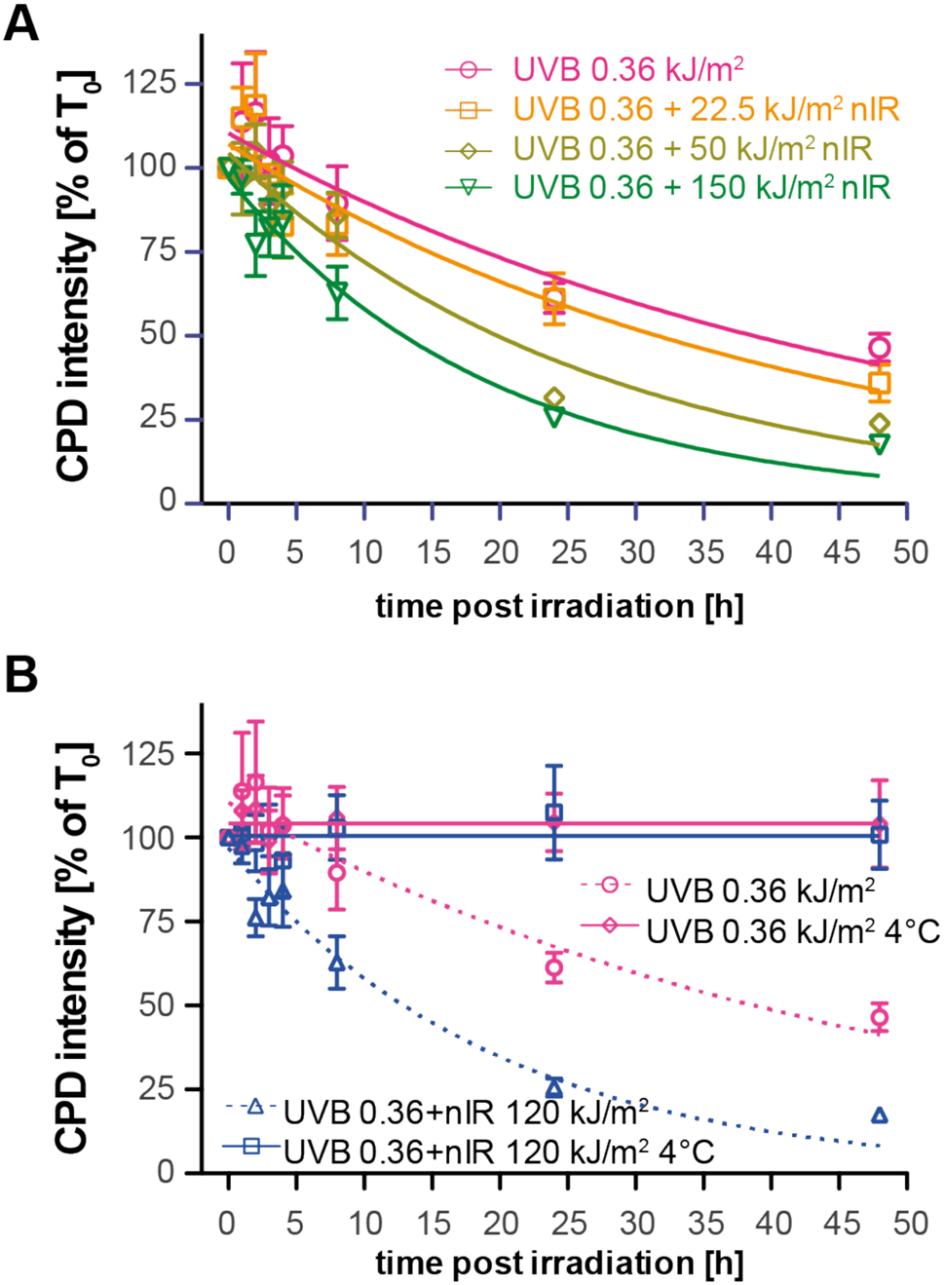
CPD removal is dependent on the nIR dose and on incubation temperature. **(A)** Immuno-slot plot quantification of the removal of CPDs after combined UVB and nIR exposure. Cells were exposed to a constant dose of 360 J/m^2^ UVB combined with varying doses of nIR. The resulting band intensities were normalized to the band intensity directly after the exposure and fitted with a single exponential function. Plotted are the mean values of at least three independent experiments, the error bars represent the standard error of the mean. (**B)** Removal of CPDs when irradiation and subsequent incubation is carried out at 4 °C. The solid lines represent the measured band intensities again normalized to the values obtained directly after irradiation. No decline in band intensity and no effect of co-exposure with nIR can be observed. The data points show the mean values of at least three replicates and the error bars represent the standard error of the mean. Curves are fitted single exponential decay curves.

Next, we systematically investigated whether the effect of faster CPD removal in the presence of nIR is dose-dependent. Again, we exposed asynchronous growing HaCaT cells to a constant dose of UVB (360 J/m^2^), while the simultaneously applied nIR dose was increased from 22.5 to 150 kJ/m^2^. Genomic CPD levels were analyzed up to 48 h post irradiation and the amount of CPDs was measured by genomic slot blot analysis. Data were again normalized to the corresponding time point directly after exposure (Fig. 2A). While the lowest nIR dose (22.5 kJ/m^2^) did only slightly reduce the CPD half-life time (from Ƭ = 33.9 h in UVB irradiated cells to Ƭ = 28.8 h in the co-exposed cells), the higher nIR doses resulted in a significant reduction of the half-life time up to Ƭ = 18.6 h (40 kJ/m^2^) and Ƭ = 13.5 h (150 kJ/m^2^), respectively. Thus, the CPD removal is faster in cells co-exposed to nIR and this effect is dose-dependent.

To further understand the underlying mechanism(s) for the observed dose-dependent decrease in CPD repair time constants, we performed irradiation experiments at 4 °C (instead of 37 °C), and also kept the temperature at 4 °C during the following 48 h. We reasoned that a pure physical process would be independent of temperature, while biological processes required physiological temperatures. HaCaT cells were exposed to either 360 J/m^2^ UVB alone or UVB in combination with 150 kJ/m^2^ nIR. Of note, when irradiation and post irradiation incubation was performed at 4 °C, no removal of CPDs was detected (Fig. 2B), neither for UVB alone nor for the combination of UVB and nIR. These findings reinforce that biological processes are the source for accelerated CPD removal in nIR co-exposed cells (see Fig. 2A).

### HaCaT cells are repair proficient

To exclude that the effect of nIR induced accelerated CPD removal is due to impaired DNA repair in the used HaCaT cells we tested whether HaCaT cells are proficient in DNA repair pathways. To study this, we exposed HaCaT cells as well as normal human dermal fibroblasts (NHDF) to a single UVA dose of 240 (HaCaT) or 120 (NHDF) kJ/m^2^ and quantified the formation of DNA repair induced foci one hour post exposure. 53BP1 and pATM showed an increase in focus formation, while γH2AX and PAR levels were found not to increase significantly (Figure S2A-D). Additionally, we compared the expression profiles of HaCaT cells with normal human keratinocytes to evaluate if significant changes in chromatin remodeling, repair or proliferation-associated genes could be identified. We compared HaCaT expression data previously generated in our lab^46^ with a dataset for normal human keratinocytes available in public databases (GEO, Accession number SE271501). Although a substantial number of transcripts are differentially expressed in the two cell types (*n* = 1,098, with log fold change >|2| and adjusted p-value < 0.05) we found only a small number of genes from the above-mentioned biological functions to be significantly different (Figure S2E and Supplementary Tables 1 and 2). We then analyzed GO functional pathway and did not find significant changes in chromatin remodeling nor DNA repair, but in proliferation, as expected from transformed versus normal keratinocytes (Figure S2F). Taken together, these data suggest that the HaCaT cells are repair proficient and show chromatin remodeling comparable to normal human keratinocytes.

### Chromatin compaction affects CPD removal

**Fig. 3:**
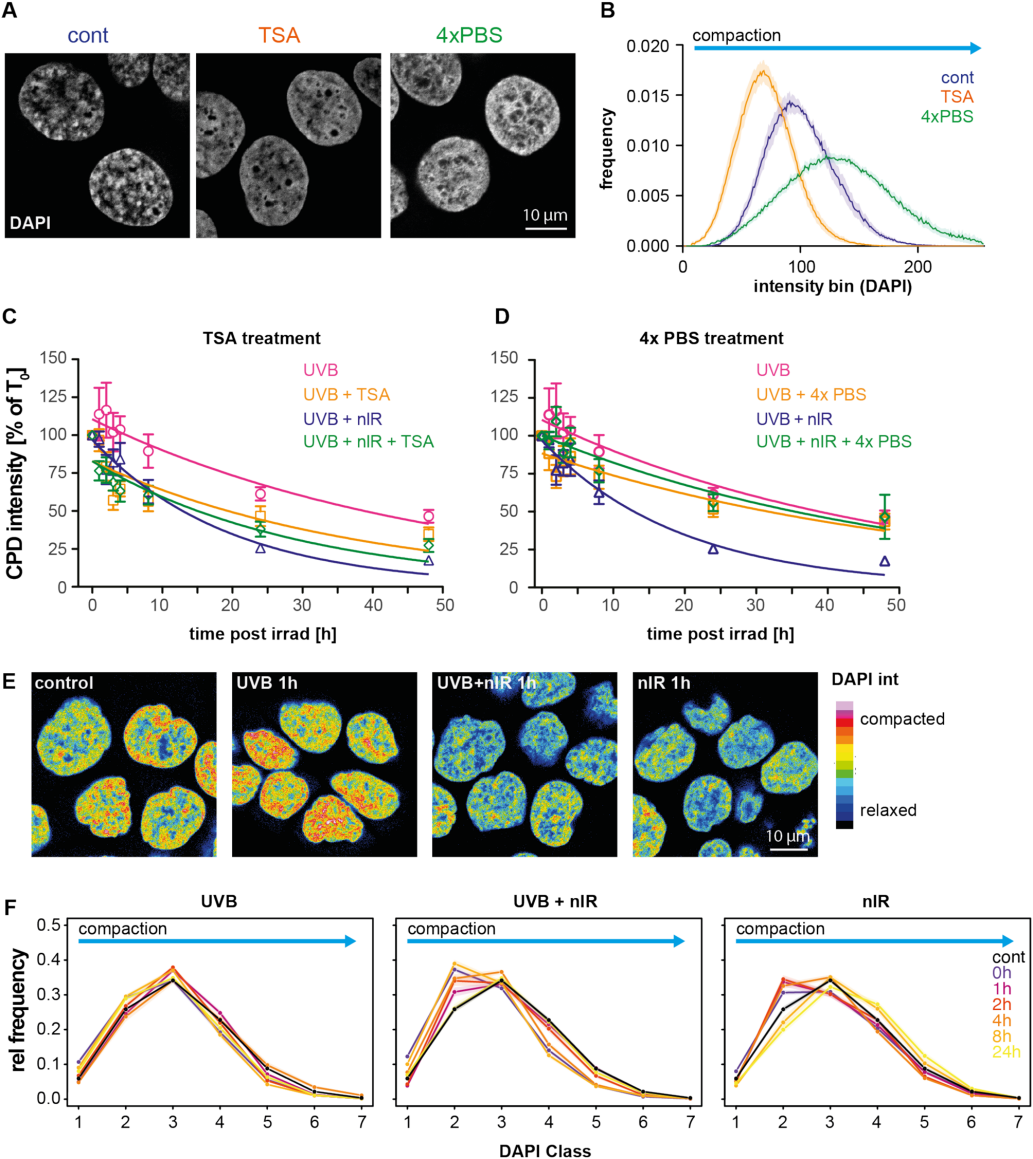
Changes in chromatin structure induced by chemical treatment or irradiation affects CPD removal. **(A)** Sample images of HaCaT nuclei, stained by DAPI and treated with either TSA or 4x PBS. (**B)** Quantification of the chromatin compaction state by the intensity histograms of the DAPI stained cell nuclei. TSA treatment (orange) induces an enrichment in the dim DAPI intensity bins compared to controls (blue line), while 4xPBS treatment enriched the DAPI bright (compacted) fractions (green line). (**C)** Effects of TSA treatment on the CPD removal after exposure to UVB or the combination of UVB and nIR. TSA pretreatment enhanced the removal of CPD removal similar to the nIR co-exposure (orange vs. pale green line). Combination of TSA and nIR did not additionally affect the CPD removal (dark green). (**D)** Artificial compaction of chromatin by 4xPBS treatment eliminated the faster removal of CPDs seen with nIR co-exposure, but did not significantly slow down the removal of CPDs when combined only with UVB exposure. (**E)** Effect of UVB, nIR and the combination of UVB and nIR on chromatin compaction. Sample images of confocal mid nuclear sections, represented in a false colour lookup table to highlight the differences. (**F)** Quantification of changes in the DAPI intensity distribution at time points up to 24 h. DAPI intensities were divided in 7 equal relative classes. Lines represent the means and the shaded areas the extend of the standard error of the mean. The datapoints show the mean values of at least three replicates and the error bars represent the standard error of the mean. Curves are fitted single exponential decay curves.

A cell biological explanation of faster CPD removal after UV-irradiation in combination with nIR might be linked to changes in the underlying chromatin structure. To test this, we artificially altered chromatin structure by means of two established treatments: first, we induced relaxation of chromatin by treatment with the HDAC inhibitor Trichostatin A (TSA). Second, we artificially compacted chromatin by means of hypertonic treatment of cells in 4x PBS, known to induce reversible chromatin compaction^47^. We quantified the chromatin compaction by measuring DAPI intensity histograms in cells following these treatments (Fig. 3A and B). As expected, treatment with TSA led to a more homogeneous DAPI distribution with lower pixel values on average, while 4xPBS treatment induced a compacted state in large proportions of the chromatin, reflected by higher DAPI intensity values (Fig. 3B yellow vs. green line).

We then irradiated pre-incubated HaCaT cells (TSA or 4x PBS) with either UVB (360 J/m^2^) alone or with a combination of UVB and nIR (360 J/m^2^ + 150 kJ/m^2^). Again, we quantified CPD amounts by immuno-slot blots for up to 48 h post exposure. Figure 3C shows the effect of TSA induced chromatin relaxation on the persistence of CPDs in genomic DNA. TSA-treated cells (yellow line) showed a faster removal of CPDs after UVB exposure compared to untreated cells (pink line). The half-life time of CPD was reduced from Ƭ = 33.9 h (untreated cells) to Ƭ = 26.6 h (TSA-treated cells). When TSA pretreated cells were irradiated with the combination of UVB and nIR (green line) the half-life time was found to be Ƭ = 20.7 h, while the combined irradiation of untreated cells resulted in Ƭ = 13.5 h. Of note, the reduction in repair decay time was in the same order of magnitude for the combination of UVB and nIR without TSA pre-incubation (orange line). This suggests that the combination of UVB and nIR is inducing chromatin relaxation that is not affected further by TSA treatment.

When we in contrast artificially compacted the chromatin by pre-incubation and irradiation in 4xPBS (Fig. 3D) we measured an increase of the half-life time of CPDs for UVB (pink line vs. yellow line) exposed cells from Ƭ = 33.9 to Ƭ = 38.5 h, indicating a reduced ability of the cell to detect the CPDs in the artificially compacted chromatin. When the cells were co-exposed to UVB and nIR pre-incubation in 4xPBS (green line) an even more pronounced increase in the half-life time from Ƭ = 13.5 h to Ƭ = 35.4 h (Fig. 3D, pink curve) was detected. This indicates that the nIR effect can be counteracted by the pre-treatment with 4xPBS.

### nIR and nIR-co-exposure causes chromatin relaxation

The above experiments suggested that nIR irradiation might itself cause chromatin relaxation, thereby allowing accelerated CPD recognition. To address this, we exposed cells either to 360 J/m^2^ UVB, to a combination of UVB (360 J/m^2^) and nIR (150 kJ/m^2^), or to 150 kJ/m^2^ nIR alone. At indicated time points post irradiation (0–24 h) we fixed the cells and stained them with DAPI. Cells were fixed in formaldehyde to preserve the 3D structure. Confocal mid nuclear Sect. (1 Airy unit) were recorded and analyzed for the DAPI intensity distribution and the pixel intensities were assigned to 7 different intensity classes according to^48^. Based on the relative pixel intensity values normalized for each cell nucleus, each pixel was assigned to one of seven classes (low DAPI intensity pixels represent less densely packed euchromatin and are assigned to class 1, while in contrast DAPI bright pixels represent compacted heterochromatin and are assigned to class 7). Figure 3E shows false colored sample images of DAPI nuclei and Fig. 3F the temporal change of the intensity values after the three different exposures scenarios. While UVB exposure alone (3F left) did not induce significant changes in any of the measured time points as compared to the non-exposed cells (black line, control), the combination of UVB and nIR exposure increased the fraction of pixels with lower relative DAPI intensity (de-compacted chromatin) especially in intensity class 2 (Fig. 3F middle). This effect was detectable directly after exposure and persisted up to two hours. Later the DAPI intensity distribution returned to a distribution similar to that of the control cells. Also, in cells exposed exclusively to nIR irradiation only, (Fig. 3F right) a similar effect was found with an increase in the chromatin class 2, reflecting an increase in decondensed chromatin. Again, in these cells we detected a return to the original distribution four hours after exposure, implying the transient nature and reversibility of the effect.

To exclude that nIR-dependent chromatin relaxation was specific for HaCaT cells, we additionally investigated human dermal fibroblasts and normal keratinocytes (Figure S3). For the fibroblasts, we found a larger fraction of compacted chromatin in unirradiated cells. Irradiated with UVB alone caused a transient increase in compacted chromatin directly after irradiation, while further changes in chromatin re-organization could not be observed. In contrast, when the fibroblasts were exposed to the combination of UVB and nIR we found a fast, transient compaction followed by relaxation (increase in DAPI classes 3 and 4) in the first hours post exposure. nIR exposure alone induced a transient de-condensation in chromatin lasting several hours post irradiation, while compaction was not detected. Normal keratinocytes showed less condensed chromatin in controls. Exposure to UVB increased the compaction in the first hours post exposure, comparable to the results seen in fibroblast, but not observed in the HaCaT cells (compare Fig. 3F). This compaction in normal keratinocytes lasted longer as compared to fibroblasts but was seen mostly in the chromatin classes 5 and 6 (Figure S3B). When UVB was applied together with nIR we also observed an increase in chromatin compaction directly after exposure, followed by a transient loss in compaction during repair time (up to 4 h post irradiation, increase in class 2). In nIR exposed keratinocytes we found no increase in compaction, but an increase in de-condensed chromatin (class 2). Chromatin returned to the control state 24 h post irradiation. Taken together, nIR-induced chromatin relaxation can be found in normal fibroblasts and keratinocytes, but they show in addition an early, transient chromatin compaction when UVB was applied simultaneously with nIR, something not seen in HaCaT cells. The UVB-related chromatin compaction followed by a reorganization during repair is in line with recent reports^49^.

To further analyse the effect of nIR exposure on the chromatin structure we performed quantitative staining of histone H3 and H4 pan acetylation (H3ac and H4ac, respectively), since acetylated histones are associated with chromatin relaxation. In line with the chromatin relaxation seen by the DAPI distribution analysis, we found that nIR exposure increased the levels of H3ac levels, directly after exposure (Figure S4A-B). The increase in H3ac levels was seen directly after irradiation (maximum 2 h post irradiation with a median increase of 1.30 fold) and persisted up to 4 h post irradiation. Then the levels returned to control levels and increased again 48 h post irradiation. In cells exposed to UVB alone, we detected a mild decreased in H3ac levels only directly after exposure. In cells exposed to the combination of both UVB and nIR we also found only a transient increase in H3ac levels, directly after exposure and a return to control levels after 4 h. The maximal increase (1.22 fold) occurred directly after exposure (0 h). Similar to the effect found for the DAPI intensity distributions (Fig. 3F), also histone acetylation showed transient and reversible chromatin relaxation. In contrast to H3ac we did not find significant changes in the levels of H4 acetylation (Figure S4C). Despite the fact that variability of the H4ac levels was generally larger in UVB-exposed cells, we could neither find a transient nor persistent increase in H4ac levels under the tested irradiation conditions.

### nIR-co-exposure causes more single strand breaks during CPD repair

**Fig. 4:**
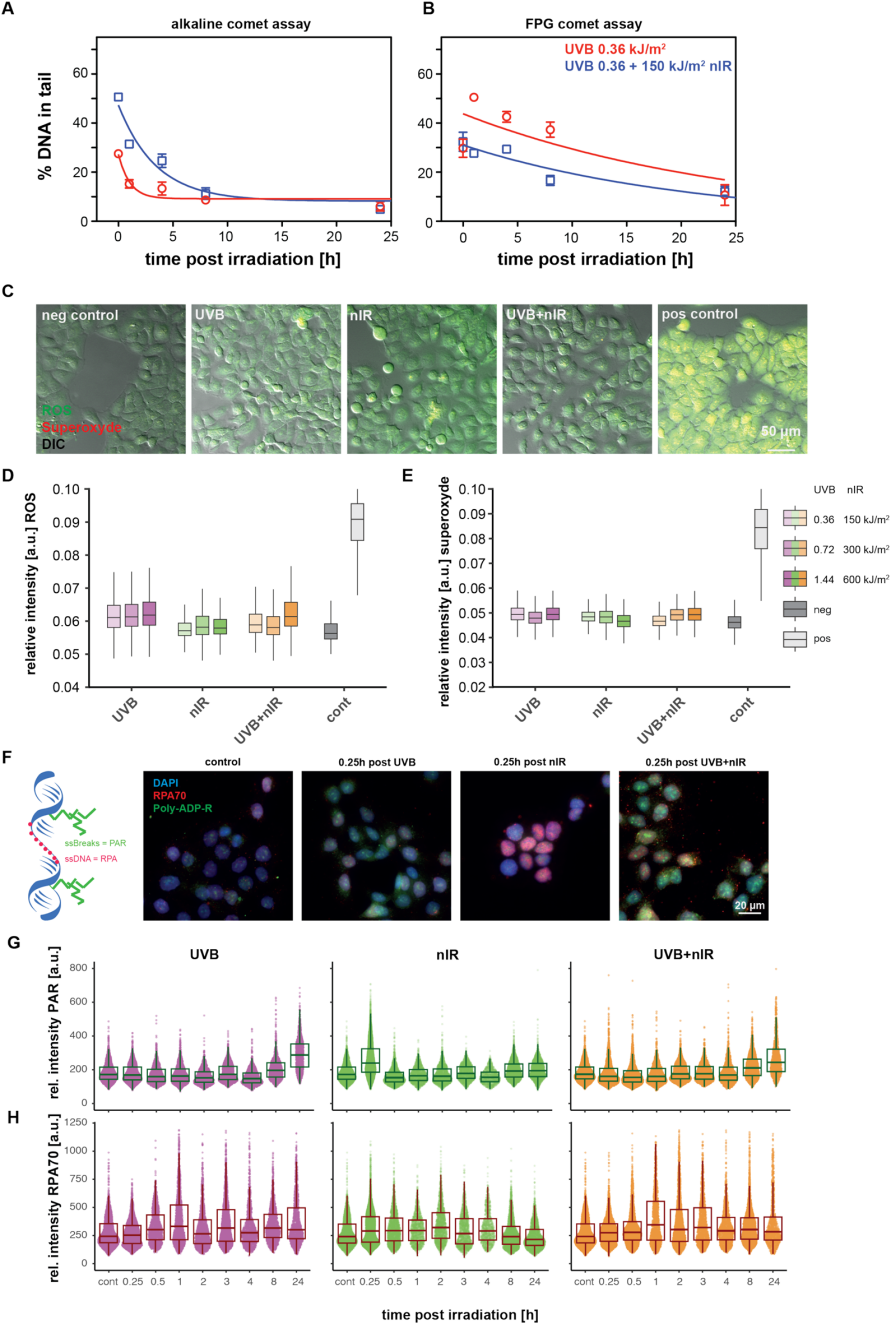
Co-exposure of UVB with nIR leads to early strand breaks without detectable ROS formation. **(A)** Alkaline comet assay quantification of strand breaks. Co-exposure (blue line) leads to initial higher levels of strand breaks. (**B)** FPG modified comet assay, shows that oxidized bases are not induced in co-exposed cells. Points represent the median of at least three biological replicates. The error bars represent the standard error of the mean. Lines are single exponential fit functions. (**C)** Detection and quantification of ROS and superoxide in UVB, nIR, and co-exposed cells. Sample images of HaCaT cells exposed to either UVB, nIR or the combination of UVB and nIR, as well as the positive and negative control. Green fluorescence indicates the ROS formation, red fluorescence the superoxide levels. (**D)** Quantification of ROS levels after 3 doses of UVB (360–1440 J/m^2^, purple), 3 doses of nIR (150–600 kJ/m^2^, green) and the combination of both (orange). (**E)** Similar to (D), the quantification of superoxide with the same cells. (**F)** Detection of single strand breaks by means of Poly-ADP-ribose (green) and single stranded DNA by means of RPA70 (red). Sample images of HaCaT cells 0.25 h post exposure to either UVB, nIR or the combination. (**G)** Quantification of poly-ADP-ribose up to 24 h post irradiation either after UVB, nIR or UVB + nIR exposure. (**H)** RPA70 intensities per nucleus analogue to G. Boxplots represent the Q1 to Q3 distribution and whiskers represent 1.5 times the IQR data from three independent replicates. In F and G, individual cell measurements are shown as dots and the resulting boxplots as described above.

Based on these results we hypothesized that the nIR co-exposure induces chromatin relaxation and thus improves the recognition of CPDs. By allowing a faster recognition and potential incision of the damaged DNA strand by the XP nucleases (XPG and XPF/ERCC1) the cells would produce repair intermediates of single stranded DNA and single strand breaks. If this step is accelerated due to altered chromatin structure, we hypothesized that the subsequent gap synthesis and re-ligation are likely to be the rate limiting steps. Thus, the cells should accumulate single stranded patches that give rise to a lower cellular survival and higher mutagenicity. To test this hypothesis, we used the alkaline version of the comet assay to quantify the levels of single strand breaks. Figure 4A shows the time-dependent quantification of single strand breaks and alkali labile sites following either exposure to UVB or the combination of UVB and nIR. Initially, we detected higher levels of DNA fragmentation in co-exposed cells (blue line) as compared to UVB only irradiated cells (red line). Values directly after irradiation were significantly higher in co-exposed cells with 45.9% DNA in tail as compared to 22.2% DNA in tail for cells exposed to UVB only. The decrease of DNA fragmentation was again faster in co-exposed cells with half-life times of 3.9 h as compared to 6.7 h for UVB alone. The alkaline version of the comet assay detects oxidative damage repair intermediates (and incisions due to base or nucleotide excision repair (BER and NER)) as well as alkali labile sites and single strand breaks. Therefore, we used the FPG modified comet assay to measure the level of oxidative bases (e.g. 8-oxoG) to exclude oxidative damage induced by the nIR spectrum (Fig. 4B). We did not find higher levels of FPG sensitive sites in co-exposed cells. Rather, the co-exposed cells showed lower levels of FPG sensitive sites compared to UVB alone (Fig. 4B, blue line), although with similar decay slopes. We further verified that ROS and superoxide are not involved in the induction of DNA fragmentation in early time points after co-exposure. Therefore, we used the ROS-ID kit, a mixture of two dyes, to detect either ROS or superoxide. Figure 4C shows sample images of HaCaT cells exposed to UVB, nIR or the combination thereof as well as an untreated control and a positive control treated with Pyocyanin. ROS formation is visible as green fluorescence, while superoxide is visible as red fluorescence. Figure 4D shows per cell quantification of the ROS levels after three different UVB doses (360-1,440 J/m^2^), as well as three different nIR doses (150–600 kJ/m^2^), and three combinations of UVB and nIR. While UVB exposure resulted in a weak increase in ROS levels at all doses, nIR did not show increase in ROS formation. The combination of UVB and nIR resulted in an increase only at the highest dose, comparable to that of UVB alone. For superoxide (Fig. 4E) we did not find significant increases neither after UVB nor nIR exposure or the combination of UVB and nIR. Therefore, we conclude that nIR does not induce significant levels of ROS nor superoxide that would result in oxidative damage, at least not under the tested irradiation conditions.

We then aimed to unravel whether UVB, nIR or the combination of UVB and nIR resulted in detectable single strand breaks and single stranded DNA. Therefore, we made use of poly-ADP-ribose (PAR), synthesized by PARP at sites of single strand breaks^50^ and the single strand binding protein RPA. We irradiated HaCaT cells as described above with a single dose of 360 J/m^2^ UVB, 150 kJ/m^2^ nIR and the combination of UVB and nIR and stained the cells for the two factors up to 24 h post irradiation. Figure 4F shows sample images of HaCaT cells stained for PAR (green) and RPA70 (red). PAR intensity in UVB exposed cells only increased four hours post exposure and showed a strong increase at 24 h post irradiation, probably indicating the onset of apoptosis (Fig. 4G left panel). Strikingly, we found a significant and transient increase of PAR signals 15 min post exposure to nIR (Fig. 4G middle panel). This coincided with high levels of ssDNA breaks as detected in the alkaline comet assay (Fig. 4A). Then PAR intensities returned to control levels and did not change further. This demonstrates a rapid and transient PARP activation upon nIR irradiation. Upon combination of UVB and nIR we only saw the UVB induced PAR signals 8 and 24 h post exposure (Fig. 4G right panel). Levels of ssDNA increased in UVB-exposed cells 30 min after exposure and increased further until one hour post irradiation (Fig. 4H left). In cells exposed to nIR only we also found an increase in the levels of ssDNA as detected by RPA70 binding (middle panel, Fig. 4H). Also in cells exposed to the combination of UVB and nIR we detected a strong increase in the RPA70 levels one hour post irradiation and the increased level persist until three hours post irradiation (Fig. 4H right). This indicates that both UVB and nIR irradiation lead to ssDNA, and the combination of UVB and nIR has an additive effect.

Taken together, in an autochthonous and ROS-independent manner, nIR induces a rapid though transient increase in ssDNA breaks and we hypothesize that this contributes to relaxation of the chromatin and thus facilitates the detection of CPDs in the chromatin.

### UVB and nIR co-exposure result in an elevated mutation rate

**Fig. 5:**
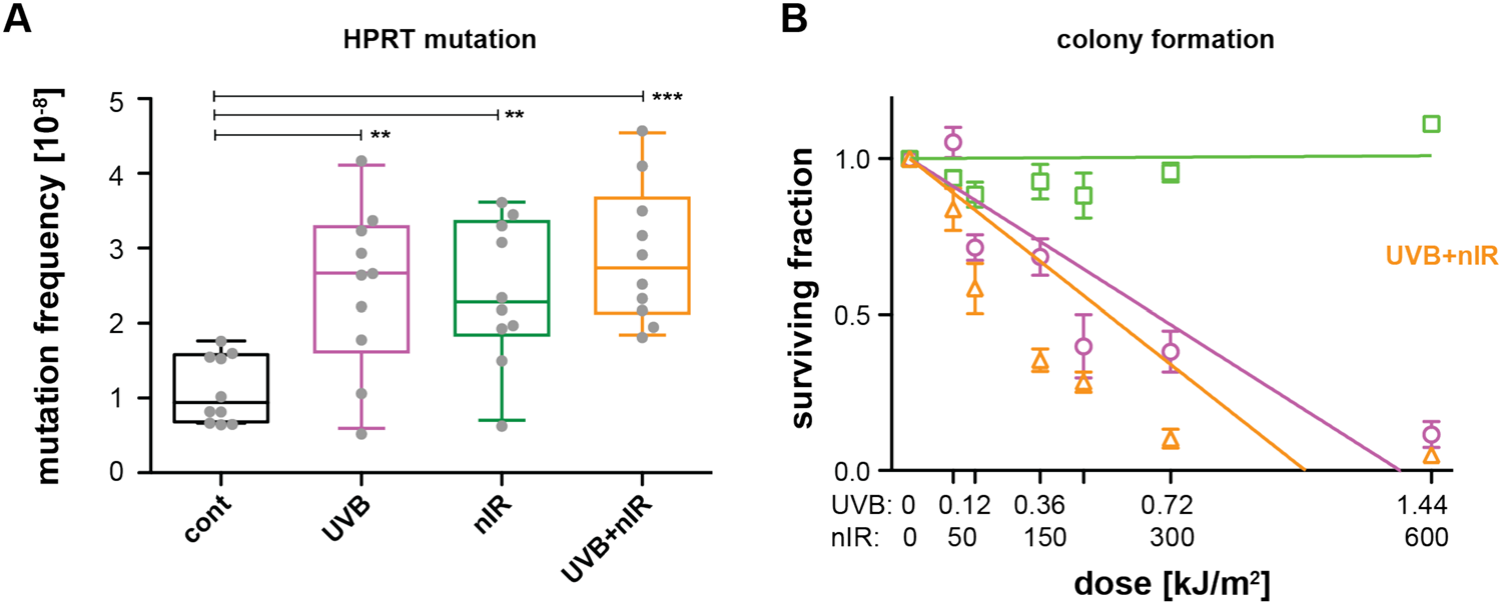
Colony formation and HPRT mutations after UVB, nIR and the combination of UVB and nIR in HaCaT cells. **(A)** Mutation frequency assessed by the colony formation after exposure of the cells to HAT medium (hypoxanthine-aminopterin-thymidine). Ten plates per irradiation condition were scored (grey dots) and are represented as box plots. Stars indicate significant differences using t-tests statistics: ** *p* < 0.01; *** *p* < 0.0001. (**B)** Colony formation assay for UVB (purple), nIR (green) and the combination (orange) for a dose range of up to 1.44 kJ/m^2^ UVB and 600 kJ/m^2^ nIR. All data points are normalized to the time matching controls. Points represent the mean of at least 5 plates from three biological replicates. Error bars represent the standard error of the mean. The data points were fitted with a linear regression.

The induction of ssDNA breaks suggests an increased mutation frequency. We therefore studied the mutation frequency induced by the different radiation regimes. Mutation frequency was assessed by the HPRT mutation assay (Fig. 5A). Again, we exposed HaCaT cells either to a single dose of 0.36 kJ/m^2^ UVB, 150 kJ/m^2^ nIR or the combination of UVB and nIR. Both, UVB as well as nIR irradiation led to a significant 2.8-fold and 2.4-fold increase in the mutation frequency, respectively. The strongest increase was seen in cells exposed to the combination of UVB and nIR, causing a 2.9-fold increase in the mutation frequency. Together, these data highlight an increased genotoxicity when UVB is combined with nIR, although the difference between combination of nIR and UVB was not significantly higher than that of UVB alone.

### Chromatin decompaction and faster CPD clearing does not benefit cellular survival

We expected that the effect of relaxing chromatin and allowing for faster CPD recognition and removal when the cells were co-exposed to nIR would be beneficial also for cell survival. Therefore, we performed colony formation assays following either UVB (180–1,440 J/m^2^), nIR (150–600 kJ/m^2^) or a simultaneous UVB and nIR exposure. Contrary to our expectations and in line with our previous findings^43^ we found that irradiation with the combination of UVB and nIR significantly reduced the number of colonies. When cells were exposed to nIR alone, we did not find a significant reduction in colony formation (Fig. 5B green line). When cells were exposed to 360 J/m^2^ UVB we found on average 68% of the corresponding controls forming colonies. When the same dose of UVB was combined with 150 kJ/m^2^ nIR the survival fraction dropped to 39%. The same trend was observed when higher doses were applied, e.g. at 720 J/m^2^ UVB the survival fraction was 38%, while in combination with 300 kJ/m^2^ nIR survival dropped to 13%. A linear regression for both exposure scenarios resulted in a slope of -0.0007 for UVB alone while the combination of UVB and nIR resulted in a slope of -0.0009. Together, this demonstrates that, although CPDs are faster detected and cleared from genomic DNA when the cells are exposed to a combination of UVB and nIR cellular survival is not increased. On the contrary and taking into consideration that nIR alone did not show any significant effect on cell survival, the combination of UVB and nIR had a super-additive negative effect on cell survival and with that on colony formation.

The cytotoxic effect of CPDs is the stalling of the replication machinery. When a DNA polymerase encounters a CPD it gets stalled and the progression of the replication fork is impaired^51,52^. One effect of stalled replication forks is the phosphorylation of H2AX (Ser 139) by the checkpoint kinase ATR or in the case of breaks, by DNA-PKcs^53^. Another cellular response to DNA damage is the formation of poly-ubiquitin chains, involved in multiple DNA repair processes and chromatin remodelling^54–56^. We therefore tested whether UVB and nIR exposure, as well as the combination affected the formation of γH2AX and the ubiquitination level in HaCaT cells. Figure S5A shows sample images of immunostaining of HaCaT cells 2 hours post irradiation with either 360 J/m^2^ UVB, 150 kJ/m^2^ nIR or the combination of UVB and nIR. We found an increase in γH2AX levels after UVB exposure, but only at later time points (2–8 h). The distribution of the measurements suggests two distinct populations. The elevated one reflects possibly S-phase cells (Figure S5B, left graph), while the lower population is probably due to cells in G1 or G2 phase. In contrast, nIR exposure did not result in changes in γH2AX levels (Figure S5B, middle). The combined irradiation of UVB and nIR resulted in effects similar to the results of UVB alone (Figure S5B, right graph). This suggests that nIR exposure has no effect on the γH2AX formation. In previous studies we analysed the effects of irradiation on the cell cycle distribution^43^ of HaCaT cells. We demonstrated that nIR exposure alone did not result in significant alterations in the cell cycle distribution (up to 72 h post irradiation), while UVB exposure as well as the combination of UVB and nIR lead to a significant S phase arrest 12 h post irradiation. The nuclear levels of ubiquitin showed a fluctuation after UVB exposure, while nIR did not show changes in ubiquitin levels. Again, co-exposure of UVB and nIR resembles that of UVB alone. These data suggest that the observed nIR effect on cellular survival is independent of γH2AX formation or protein ubiquitination.

## Discussion

Rather than using narrow banded UV light sources, we here assessed CPD induction and repair in the context of the individual and combined spectral bands (UVB, UVA VIS and nIR) and with that more closely related to the situation of environmental sun light. When we studied the effect of combinational exposure, we found that UVB and UVA have a rather additive effect on the induction of CPDs, with UVB accounting for the majority of the induced CPDs in accordance with previous reports^6^. When we combine UVB as well as UVA with near infra-red (nIR) upon simultaneous exposure, we unexpectedly found reduced induction of CPDs and/or a faster clearing of these DNA lesions. As previously described, nIR does not result in direct CPD formation^57^although some studies report formation of ROS^58^when using VIS and nIR lasers or high energy densities^32^. When we quantified the effect of nIR on CPD removal we found that the effect is dose-dependent and is also temperature sensitive. A thermal effect from the nIR irradiation could be excluded, since the nIR source is water filtered and actively cooled. Similarly, the temperature of the cells and culture dishes was controlled during irradiation and the temperature increase during irradiation remained below one degree Celsius.

Both induction and repair of the CPDs are dependent on chromatin structure^9^ and characteristic “photofootprints” can be detected. Chromatin compaction changes as well as changes in the epigenetic marks following UV exposure have been reported before. Accordingly, reorganization of H3K27ac decorated chromatin following UVC exposure^59^ or fast large scale chromatin reorganization^8,60^ have been shown. Here we demonstrate that also nIR exposure causes chromatin changes, namely relaxation in a temporal and transient manner. Previously it was suggested that nIR exposure causes chromatin “vibration”^36^ and protective effects of nIR have been reported in several studies^35,61–64^. Potential mechanisms include pre-sensitizing of cells through low levels of ROS, SOD activation, and anti-apoptotic effects^61–64^. Similar effects have also been reported when cells were pre-exposed to nIR before X-ray exposure^37^. Local chromatin remodelling is an essential step in gg-NER, when either photo-lesions require to be made accessible from within the nucleosome structure or the nucleosomes in the vicinity of the damage need to be shuffled or removed during the repair process^10,23^. One can imagine that the nIR induced chromatin “vibration” enhances the local chromatin remodelling and therefore accelerates/promotes damage recognition by the UV-DDB complex. This might also affect local chromatin ubiquitination and nucleosome eviction^65^ as well as the subsequent NER steps^66^.

Interestingly and in contrast to the above, we find that the cellular outcome of the combined exposure of UVB and nIR did not have beneficial effects on cell survival and that the mutation rate increased compared to controls and UVB exposure alone. Therefore, we argue that the nIR- induced chromatin relaxation allows for CPDs to be recognized faster, probably through the gg-NER sub-pathway, where damage recognition is rate-limiting. If damage recognition, followed by the verification and incision^19,67–69^ were accelerated, the subsequent re-synthesis and re-ligation would be the rate-limiting step^70^. In this case, cells would accumulate elevated levels of single strand breaks and single stranded DNA patches. This would provide the cause for the observed elevated mutation rate and reduced survival due to co-exposure of nIR with UVB. This model would also explain the elevated levels of single strand breaks observed after simultaneous UVB with nIR exposure.

Taken together, we demonstrate an altered induction and repair kinetic of CPDs in the presence of nIR, with chromatin relaxation being at least one reason for its faster recognition. It is worth mentioning that the faster recognition is not beneficial for the cell and the fidelity of the repair process. Since the removal of CPDs from genomic DNA in this case is not a proper “DNA repair” we rather call this process “CPD removal”. Therefore, risk estimation should be based on environmentally relevant irradiation conditions rather than artificial radiation sources. Whether the described effects are also relevant for the situation in sun-exposed human skin, remains to be seen.

## Methods

### Cell culture

HaCaT cells^71^ were cultivated in DMEM (4.5 g/L Glucose, L-Glutamine, Sodium pyruvate, 3.7 g/L NaHCO_3_) (Pan Biotech) containing 10% FCS and 0.1% Pen/Strep (Pan Biotech) at 37 °C in the presence of 5% CO_2_. Cells were verified by ATCC STAR authentication. We on purpose chose the spontaneously immortalized, non-tumorigenic HaCaT cells (70). Similar to many normal keratinocytes in human UV-exposed skin, and the potential precursor cells for skin cancer, HaCaT cells contain UV-indicative p53 mutations and cSCC-characteristic genetic alterations. Nevertheless the HaCaT cells, as their counterparts in human skin are not tumorigenic and under optimal conditions are still competent for epidermal differentiation, i.e. to form an epidermis-like epithelium when propagated as skin equivalents in 3D organotypic cultures^72^. Accordingly, HaCaT cells present a valuable model commonly used for this type of studies.

TSA treatment was performed for 24 h at a concentration of 0.25 µM prior to irradiation in complete medium. For irradiation the medium was replaced with pre-warmed PBS as described below. Repair was performed in un-substituted media. For the 4xPBS treatment cells were rinsed three times in 4x PBS and then pre-incubated for 10 min at 37 °C in 4x PBS. Irradiation and was carried out in 4xPBS and then cells were kept again in regular growth media. Human keratinocytes (HNEK) and dermal fibroblasts were isolated from surgery material with written consent from the patients. For subculturing keratinocytes were seeded on collagen (human placenta type IV, Sigma Aldrich) coated dishes or slides.

### Irradiation

Cells were seeded in the indicated culture vessels and irradiated in pre-warmed PBS with the indicated doses and spectral combinations. During irradiation the temperature of the cells was kept constant by the built-in Peltier temperature device (Figure S6A). The spectral details of the irradiation device are described in^43^. In short, UVB irradiation as delivered with a dose rate of approx. 0.47 J/m^2^/s. UVA with a dose rate of 38.5 J/m^2^/s VIS with 215 J/m^2^/s and nIR with 250 J/m^2^/s. Since cells were irradiated for a prolonged time (up to 50 min) with an open lid, we measured the evaporated PBS value during the irradiation process. The total evaporation was less than 10% in the case of a one hour evaporation with all lamps simultaneously switched on (Figure S6B).

In order to access the effect of different spectral ranges on cells we measured the absorbance of cultured cells. Since the absorption of a single cell layer is very small we stacked ten completely confluent coverslips of HaCaT cells and measured the irradiance of the complete spectrum (250-1,000 nm) in comparison to 10 coverslips alone used as blank by using a calibrated spectrometer (specbos 1211 UV, Jeti Instruments, 100 times averaging, per measurement, 10 measurements averaged). The irradiance measurements were converted to spectral absorption and the integrals of the four spectral ranges were calculated (Figure S6C). Data for quantum yield (Φ), for CDP formation have been summarized by Cadet and Douki^73^ for irradiated cell cultures as well as for human skin explants for different wavelength regions (UVC, UVB and UVA). The authors report a quantum yield Φ_UVC_ for CPD formation in cell cultures in the range of 0.1-1.0 CPD/10^5^ bases per J/m^2^, yielding a mean Φ_mean, UVC_ = 0.45 CPD/10^5^ bases per J/m^2^ for UVC- (254 nm) irradiation. For UVB- irradiation the quantum yield is roughly an order of magnitude lower with a mean of Φ_mean, UVB_ = 0.05 CPD/10^5^ bases per J/cm^2^^74^. For broadband UVA-irradiation of human melanocytes much lower quantum yields of Φ_melano, UVA_ = 0.13 and for keratinocytes a mean of Φ_kerato, UVA_ = 0.1 CPD/10^10^ bases per J/m^2^ have been reported^75,76^. Broadband UVA-irradiation of human skin explants yields mean quantum yields, Φ_hum skin explants_ of about 0.13 or 0.21 (for skin type IV, II) or 0.09 (regardless of skin type) CPD/10^10^ bases per J/cm^2^. To assess the effect also for the visible and near-infrared spectrum we measured the absorbance of HaCaT cultures in the range of 250-1,000 nm (Figure S6C-D) and found the majority of absorbance in the UV range (UVB 0.09 OD units, UVA 0.27 units respectively), but also absorption in the visible range (0.46 OD units). In the limited range (750-1,000 nm) of the nIR spectrum that we could measure, we did not find significant absorption, but this might be due to the limitation of the used spectrometer.

### CPD quantification by slot blot

Total genomic DNA was extracted using QIAamp DNA Mini Kit (Qiagen) and 150 ng of DNA were diluted in 100 µL of TE buffer including 50 mM NaCl. DNA was denatured by incubation at 95 °C for 5 min, followed by quenching at 4 °C. Then 100 µL of 20x SSC (3 M NaCl, 300 mM sodium citrate) were added. The slot blot was prepared by soaking the nitrocellulose in 20x SSC buffer and the filter paper in ddH_2_O. Each well was pre-soaked with 200 µL 20x SSC before the samples were loaded and allowed to adsorb for 15 min. After washing each well with 200 µL 20x SSC, the membrane was taken out of the apparatus and soaked in 0.4 M NaOH for 15 min, followed by a short wash in 5x SSC. Then the membrane was blocked in PBS / 0.2% Tween 20 / 5% milk powder for one hour. After a short wash in 5x SSC the membrane was incubated in primary antibody solution (mouse-anti-CPD, clone TDM-2, Cosmo Bio; 1:1000 in PBS/ 0.2% Tween) for 2 h at room temperature. The membrane was washed twice as described above and incubated in secondary antibody solution (goat-anti-mouse-IgG-Cy3; Amersham, 1:5000) for 1 h at room temperature. After another two washes, images were acquired using an Imager 600 (Amersham). As loading control, the membranes were stained after CPD detection for total DNA amount by incubation in 0.2% methylene blue solution (in 300 mM NaAc, pH 5.0) and imaged using epi-illumination. Band quantification was done using ImageJ. All full blots are provided in the supplementary data.

### CPD quantification by flow cytometry

For each measurement 5 × 10^5^ HaCaT cells were seeded in 60 mm petri dishes and grown under standard conditions (see above). Cells were exposed in PBS to different doses of UVB and UVA at a confluency of 80–90%. Immediately after exposure the cells were harvested by trypsinization. Cells were fixed in 70% EtOH by drop wise addition to a concentration of 1 × 10^6^ cells/mL and incubation at -20 °C for at least 1 h. For CPD staining cells were permeabilized by treatment with 1x blocking/washing solution (BWS = 0.125% Triton X-100 in Roti Block, Roth) for 10 min at RT. For DNA denaturation cells were resuspended in 2 N HCl (Roth) for 10 min at RT. After washing the cells using BWS they were incubated in a proteinase K (5 µg/ml, Sigma) solution for 10 min at 37 °C and after another wash CPDs were labelled using 1 µg/ml mouse anti-CPD antibody (clone KTM53, Kamiya Biomedical Company) in BWS overnight at 4 °C. The next day cells were washed again and subsequently stained using 15 µg/ml goat anti-mouse-IgG FITC-conjugated antibody (Dianova) in BWS for 1 h at 37 °C. After the last wash, cells were incubated for 5 minutes in 10 µg/ml propidium iodide and 0.01% RNAse (both Sigma) in PBS at RT prior to the measurement using the Guava easyCyte 8HT flow cytometer (Merck). At least 5000 cells per sample were analysed. Data analysis was performed using Flowing Software 2.5.1 (Perttu Terho, Finland). Solely single cells in G1 phase were quantified (see Fig. 1G).

### Colony formation assay

HaCaT cells were trypsinized and seeded at a density of 50–150 cells per 35 mm dish. Cells were allowed to attach overnight before irradiation. Irradiation was carried out in prewarmed PBS as described above. Control cells for each time point were held in PBS under identical conditions for the same amount of time. After irradiation PBS was replaced with fresh media and cells were allowed to grow for 7–9 days. Then the cells were fixed in -20 °C MeOH for 15 min at room temperature and subsequently stained in 0.2% methylene blue in 50% MeOH for 15 min. Excess staining solution was removed and colonies were washed in ddH_2_O until the background was clear. Colonies were counted by a semi-automated ImageJ macro script (see supplementary material). Colony numbers of the individual irradiation doses were normalized to the counts of the corresponding mock-treated control cells.

### HPRT assay

HRPT assay was conducted according to^77^. HaCaT cells were seeded at 50% confluence in regular growth medium. The next day the medium was replaced with HAT medium (100 µM hypoxanthine, 0.4 µM aminopterin, 16 µM thymidine) to negatively select for background mutations. After 6 days in HAT medium, the cells were split and seeded in regular growth medium with either 1 × 10^5^ cells per 10 cm dish for irradiation or with 200 cells per 10 cm dish as plating control. The cells for irradiation were irradiated the next day as described above and subsequently transferred to regular growth medium substituted with 6-ThioG containing medium (40 µM). Cells were then cultured for 7 days in HAT medium following 10 days in regular medium. The control cells were grown for 15 days in regular growth medium. Then the cells were fixed in ice cold methanol and stained as described above in the section colony formation assay. The mutation frequency was calculated according to the following equation Plating efficiency (PE) = colonies formed/cells seeded × 100, mutation frequency = number of resistant colonies/(number of cells seeded × PE) × dilution factor with dilution factor = number cells seeded per plate in PE experiment/number cells seeded in mutation frequency.

### Comet assay

HaCaT cells were irradiated as described above and post incubated in complete medium under standard conditions for the indicated times. Then cells were trypsinized and embedded in low melting point agarose (Sigma Aldrich, Type VII) at a finale concentration 5 × 10^4^ cells in 0.75% agarose in PBS. Gels (50 µl) were cast directly on gelbound film in 8 well chambers (ibidi) and solidified on a cold metal plate. Lysis and electrophoresis was performed according to^78^in short, overnight lysis at 4°C was performed in lysis solution (2.5 M NaCl, 100 mM EDTA, NaOH adjusted pH to 10 and 1% Triton X100). Then gels were transferred to the precooled electrophoresis chamber filled with electrophoresis solution (0.3 M NaOH, 1mM EDTA, pH 13) and incubated for 30 min for unwinding. Electrophoresis was performed at 0.8 V/cm for 25 min. After electrophoresis the gels were immersed twice in neutralisation buffer (0.4 M Tris HCl, pH 7.5) for two times 20 min and then dehydrated in 100% EtOH for 10 min. After air drying overnight DNA was stained with SybrGold (Thermo Fisher S11494, 1:10,000 diluted in PBS) for 2 h and embedded in DABCO antifade solution.

For enzyme modified comet assays, the embedded and lysed cells were washed 3 times for 10 min each in PBS before the gels were equilibrated in the corresponding reaction buffer for 15 min (T4 endo V buffer or FPG buffer). T4 endo V (also known as pyrimidine dimer glycosylase, NEB M0308S) reaction buffer was supplemented with 0.2 mg/ml BSA and the enzyme was diluted 1:30,000. To each gel 50 µl (0.04 U) were added and incubated in a humidified chamber for 30 min at 37 °C. After incubation gels were rinsed twice in electrophoresis buffer and processed as described above. Buffer only controls were made in parallel and in the end the % DNA in tail values from the buffer only controls were subtracted from the T4 endo V treatment measurements.

For the detection of oxidative lesions FPG (formamidopyrimidine [fapy]-DNA glycosylase, NEB M0240S) was used similarly. Gels were equilibrated in FPG buffer and FPG was used at a dilution of 1:3,000 (0.3 U/gel). Again, buffer only controls were made and subtracted from the enzyme treated values. Comet images were recorded using a Zeiss Axiovert 200 microscope equipped with a Plan-Neofluar 10x NA 0.3 objective lens, a 482/18nm excitation 495 beam splitter and 520/28nm emission filter and an Axiocam mRM. Recorded images were analysed using Komet 7 (Andor). At least 50 comets were analysed from 3 independent biological replicates.

### ROS quantification

ROS and superoxide were simultaneously, detected using the ROS-ID kit (Enzo Life Science ENZ-51010) according to the manufacturer’s recommendations. Total ROS were detected with Oxidative Stress Detection Reagent using the green fluorescence and superoxide was quantified using Superoxide Detection Reagent using red fluorescence emission. In short cells were seeded and irradiated as described above with three doses. Immediately after irradiation the PBS was replaced with freshly prepared staining solution (ROS-ID green 1:2,500 and ROS-ID red 1:2,500 diluted in serum free medium and calcein blue AM (Thermo Fisher # C34853). After incubation for 45 min at 37 °C living cells were directly imaged using a Nikon Ti2 microscope equipped with a Lumencor spectra LED light source, 20x S Plan Fluor LWD 0.7NA objective and filter sets for FITC (Semrock LED-DAPI/FITC/TRITC/Cy5-4x-B) and mCherry (Semrock LED-CFP/YFP/mCherry-3X-A-000). Pyocyanin (250 µM) was used as a positive control for 30 min under regular culture conditions.

Images were analysed using CellProfiler^79^. The pipeline is available in supplementary materials.

### Immunofluorescence staining

HaCaT cells or NHEK cells were seeded on glass coverslips and either pre-extracted in 4 °C PBS + 0.5% Triton X 100 for 2 min with shaking at the indicated time points post irradiation or directly fixed. Then cells were fixed in 3.7% formaldehyde in PBS for 15 min at room temperature. Permeabilization was performed in 0.7% Triton-X 100 in PBS for 15 min at room temperature. Slides were blocked in 1% BSA (Type V) in PBS for 30 min at room temperature. Primary antibodies were diluted in PBS/1% BSA (mouse-anti-PAR, Calbiochem clone H10, 1:200; rabbit-anti-RPA70, Epitomics, 1:100; rat-anti-Histone H3, Active Motif clone 1C8B2, 1:400; rabbit-anti-H4 pan acetylation, Merck Millipore #06-866, 1:100; rabbit-anti-Histone H3 pan acetylation, Upstate # 29505, 1:200; rabbit-anti-gH2AX, Abcam #GR307808-2, 1:800; mouse anti-ubiquitin-protein, clone FK2, Upstate, 1:200; rabbit-anti-53BP1 Novus Biological #NB100-904 1:100; rabbit-anti-pATM, Abcam clone EP1890Y, 1:100; mouse-anti-gH2AX, Merck Millipore, clone JBW301, 1:100) and incubated for 2 h at room temperature. After three washes in PBS/0.025% Tween 20 slides were incubated with the secondary antibodies diluted in 1%BSA/PBS (goat-anti-mouse-Alexa488, Invitrogen #A-21241; goat-anti-rabbit-Alexa594, Jackson Immunolabs #111-585-144, both 1:750; donkey-anti-rat-IgG, Jackson Immunolabs #712-585-153, 1:800) for 1 h at room temperature. After washing as described above cellular DNA was counterstained with 1 µM DAPI in PBS for 10 min, washed once in ddH_2_O and mounted in Vectashield (Vecorlabs). Imaging was performed using a Nikon Ti2 microscope using a 40x CFI Plan Apo λ 0.95 NA objective and filters for DAPI and Alexa488 (Semrock LED-DAPI/FITC/TRITC/Cy5-4x-B) and Alexa594 (Semrock LED-CFP/YFP/mCherry-3X-A-000). Images were quantified using CellProfiler, the analysis pipeline is available in the supplementary information.

### DAPI intensity distribution

Chromatin compaction changes were measured in HaCaT cells as well as in human fibroblasts and keratinocytes (NHEK). Cells were grown on coverslips and irradiated either with 720 J/m^2^ UVB, 300 kJ/m^2^ nIR or the combination of both in prewarmed PBS. Then cells were fixed at the indicated time points in 3.7% formaldehyde in PBS for 15 min at room temperature to preserve the 3D structure. Cells were stained in 5 µg/ml DAPI solution in PBS for 15 min, rinsed quickly in ddH_2_O and embedded in mounting media. Imaging was performed using a Leica SPE confocal microscope using the 405 nm excitation laser and a detection window from 410 to 470 nm. The pinhole was set to 1 Airy unit. An ACS APO 63 × 1.3 NA objective lens was used to image mid nuclear planes. At least 12 (keratinocytes) or 25 (fibroblasts) randomly selected fields of views were recorded and only cells with recorded mid nuclear sections were analysed. Image analysis was performed in ImageJ by extracting the numerical values of each mid nuclear section and then further processed using R.

### Expression analysis

Expression data of HaCaT cells previously generated in our lab^46^ (Illumina HumanHT-12 V4.0 expression beadchip) was compared to publicly available expression data for normal human keratinocytes (GEO, Accession number SE271501). Only the control samples were taken into consideration for the analysis (GSM8378055, GSM8378056, GSM8378057). The analysis addresses the differential expression between the two cell lines (human keratinocytes were used as reference) done using Limma. Among the differentially expressed transcripts, we selected the transcripts per genes with the highest expression. Among 17,142 transcripts, we found 6,617 transcripts that were significantly (p value < 0.05) differentially expressed. Gene ontology analysis was performed using BioMart followed by GSEA to generate ethe bubble plots, and we specifically included terms containing DNA repair, chromatin and replication.

### Data analysis

Data was analysed using Graphpad Prism Version 5.0 (GraphPad Software) or R-studio (Posit). Curve fitting of repair kinetics were performed with an exponential decay with the following constrains: curves were fitted through 0 h/100% and the plateau is set to 0. Linear fitting was done with no constrains.

## Data Availability

All primary data are available from the TU datalib (https://tudatalib.ulb.tu-darmstadt.de/handle/tudatalib/4646).
